# Rapidly Progressive Joint Destruction in Idiopathic Multicentric Castleman’s Disease: A Case Report

**DOI:** 10.7759/cureus.85108

**Published:** 2025-05-30

**Authors:** Haruki Matsumoto, Naoki Matsuoka, Kazuhiro Tasaki, Noriyoshi Sato, Masahito Kuroda, Masayuki Miyata

**Affiliations:** 1 Department of Rheumatology and Collagen Disease, Fukushima Red Cross Hospital, Fukushima, JPN; 2 Department of Rheumatology, Fukushima Medical University School of Medicine, Fukushima, JPN; 3 Department of Gastroenterology, Fukushima Rosai Hospital, Fukushima, JPN; 4 Department of Pathology, Fukushima Red Cross Hospital, Fukushima, JPN; 5 Department of Orthopedic Surgery, Fukushima Red Cross Hospital, Fukushima, JPN; 6 Department of Gastrointestinal Medicine, Fukushima Red Cross Hospital, Fukushima, JPN

**Keywords:** idiopathic multicentric castleman's disease, il-6, mammalian target of rapamycin (mtor) inhibitor, rheumatoid arthritis, synovitis

## Abstract

Castleman's disease (CD) is a group of lymphoproliferative disorders characterized by common morphological features on lymph node biopsy, with idiopathic multicentric Castleman's disease (iMCD) being a notable subtype. Here, we report a 69-year-old Japanese iMCD patient complicated by rapidly progressive joint destruction. Her joint destruction progressed rapidly around the time of her iMCD diagnosis, and she underwent right total hip arthroplasty (THA). Synovial tissue revealed rheumatoid arthritis (RA)-like synovitis. Joint destruction continued to progress even after tocilizumab (TCZ) was initiated for iMCD. Before long, she underwent left THA. Joint destruction due to cytokines other than interleukin-6 (IL-6), or other inflammatory pathologies, was suspected. Mechanistic target of rapamycin (mTOR) activation in iMCD may promote synovitis and joint erosion by regulating immune cell function and osteoclast differentiation. Although there are common pathological mechanisms between iMCD and destructive synovitis (RA-like synovitis), a case of rapidly progressive bone destruction has not been previously reported. Therefore, we report a unique case of iMCD complicated by destructive synovitis (RA-like synovitis) to contribute to the understanding of these overlapping disease mechanisms and potential therapeutic strategies.

## Introduction

Castleman's disease (CD) is a heterogeneous group of lymphoproliferative disorders that share common morphological features on lymph node biopsy; idiopathic multicentric Castleman's disease (iMCD) is a subtype of CD [1]. iMCD is characterized by the involvement of multiple lymph node regions and is frequently associated with systemic symptoms, including fever, night sweats, hepatosplenomegaly, and laboratory abnormalities such as elevated inflammatory markers [1].

While musculoskeletal symptoms, such as arthralgia, may occasionally occur, inflammatory arthritis is rare and not a common symptom of iMCD [2]. Pathologically, CD is classified into hyaline vascular, plasma cell, and mixed variants. The plasma cell variant, more commonly seen in iMCD, is characterized by hyperplastic follicles and an abundance of plasma cells in the interfollicular areas [2].

Elevation of various inflammatory cytokines - especially interleukin-6 (IL-6) [3] - and abnormal activation of the mechanistic target of rapamycin (mTOR) are involved in the inflammatory pathogenesis of CD [4]. These inflammatory mechanisms may lead to other unforeseen inflammatory diseases. In fact, CD is often reported to be complicated by autoinflammatory or autoimmune diseases [5,6].

CD and rheumatoid arthritis (RA) share common inflammatory cytokines in their pathogenesis. IL-6 plays a central role in both conditions, contributing to inflammatory abnormalities [7]. Similar to CD, mTOR activation is crucial in RA, contributing to bone destruction through the regulation of immune cell function, osteoclast differentiation, and survival, ultimately promoting synovitis and joint erosion [8,9].

Despite these shared pathogenic mechanisms, reports of co-occurrence of RA-like synovitis and CD are rare, and there are no documented cases of rapid bone destruction in patients with both conditions. We describe the case of a patient with iMCD complicated by destructive synovitis, despite having no symptoms of joint pain. Even if the patient is not aware of joint pain, bone destruction may progress if inflammation persists in the pathological and immunological microenvironment. This patient had histologically proven RA-like synovitis and showed rapidly progressive joint destruction, suggesting that the inflammatory pathology of CD contributes to joint destruction.

## Case presentation

A 69-year-old Japanese woman was admitted to our department. Since the age of 63, she had been seen by a local orthopedic doctor for suspected osteoarthritis of both knees. She was not aware of any joint pain or swelling. During follow-up, anemia was diagnosed, and she was referred to our department for further examination. The laboratory findings are shown in Table 1.

**Table 1:** Laboratory findings at the time of admission in our department Ab, antibody; ANA, anti-nuclear antibody; APTT, activated partial thromboplastin time; C, complement; CCP, cyclic citrullinated peptide; CLβ2GPI, anti-cardiolipin-beta2 glycoprotein 1 complex; HBsAg, hepatitis B virus surface antigen; HCV, hepatitis C virus; HHV, human herpesvirus; -Ig, immunoglobulin; IL, interleukin; PT-INR, prothrombin time-international normalized ratio; RF, rheumatoid factor; SAA, serum amyloid A

Variable	Patient's value	Standard value
Peripheral blood		
Red blood cells	331 × 10^4^/μL	386-492
Hemoglobin	8.7 g/dL	11.6-14.8
Hematocrit	27%	35.1-44.4
Platelets	80.9 × 10^4^/μL	15.8-34.8
White blood cells	6,700/μL	3,300-8,600
Neutrophil	75%	40-75
Eosinophil	0%	0-6
Monocyte	6%	1-14
Lymphocyte	19%	20-50
Baso	0%	0-1
Blood chemistry		
Total protein	6.9 g/dL	6.7-8.3
Total bilirubin	0.3 mg/dL	0.2-1.0
Albumin	1.9 g/dL	3.9-4.9
Aspartate aminotransferase	16 IU/L	13-33
Alanine aminotransferase	11 IU/L	6-27
Lactate dehydrogenase	192 IU/L	119-229
Alkaline phosphatase	99 IU/L	38-113
Creatine kinase	69 IU/L	45-163
Blood urea nitrogen	14.8 mg/dL	8-23
Cr	0.33 mg/dL	0.45-1.24
Na	135 mEq/L	133-150
K	4.7 mEq/L	3.6-4.9
Cl	100 mEq/L	95-110
Fe	6 μg/dL	50-188
Ferritin	358 ng/mL	5-179
Immunoserological tests		
C-reactive protein	17.43 mg/dL	0-0.20
IL-6	153 pg/mL	<4.0
IgG	2,303 mg/dL	870-1,700
IgG4	636 mg/dL	11-121
IgA	287 mg/dL	110-410
IgM	144 mg/dL	46-260
M-protein	(-)	(-)
C3	138 mg/dL	86-160
C4	43 mg/dL	17-45
ANA	<1:40	<1:40
RF	32.8 IU/mL	0-15
Anti-CCP Ab	0.6 U/mL	<4.5
Anti-CLβ2GPI Ab	<1.2	0-3.6
Lupus anticoagulant	0.9	0-1.3
Soluble IL-2 receptor	855 U/mL	121-613
SAA	1,440 mg/L	0-8.1
Coagulation		
PT-INR	1.38	0-5
APTT	38 sec	23.7-34.7
Infection		
HBsAg	(-)	(-)
Anti-HCV Ab	(-)	(-)
HHV-8 DNA	<2.0 × 10 copy	<2.0 × 10
Urinalysis		
Occult blood	(2+)	(-)
Protein	(-)	(-)

**Figure 1:**
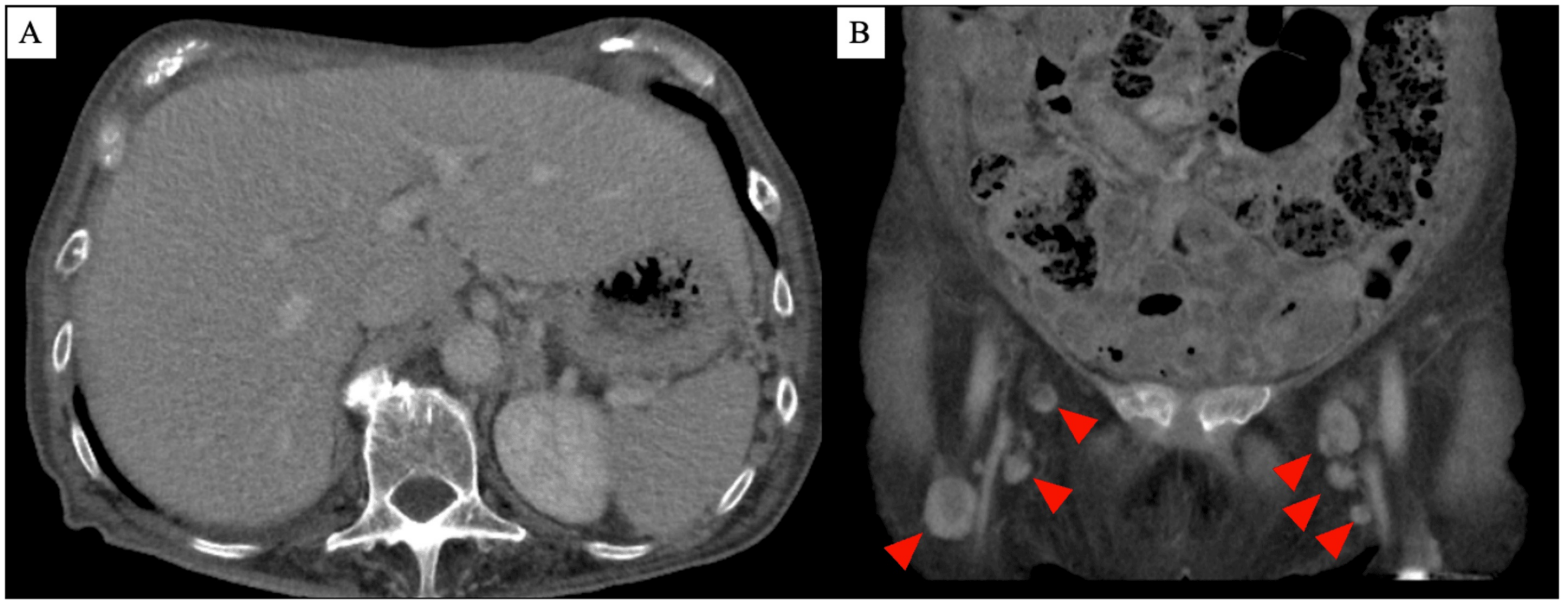
CECT of the abdomen and pelvis (A) Abdominal CECT revealed liver enlargement; (B) Pelvic CECT of the pelvis revealed lymph node enlargement in both inguinal regions (red arrowheads). CECT, contrast-enhanced computed tomography

Her blood test results showed low iron levels, high ferritin levels, and markedly elevated C-reactive protein (CRP) levels, suggesting that anemia was likely caused by chronic inflammation. Antinuclear antibodies were less than 1:40 and were not clinically significant. The level of serum anti-cyclic citrullinated peptide antibody was <0.6 U/mL, and RF was 32.8 IU/mL. Although she was under observation as an outpatient, bilateral leg edema gradually developed, which worsened over time, leading to difficulty in movement. Additionally, she had fever, weight loss, fatigue, and night sweats. Consequently, she was admitted to our department. Contrast-enhanced computed tomography showed liver enlargement (Figure 1A) and lymph node enlargement, with a maximum diameter of 13 mm in both inguinal regions (Figure 1B), but no pleural or ascitic effusions were observed.

**Figure 2:**
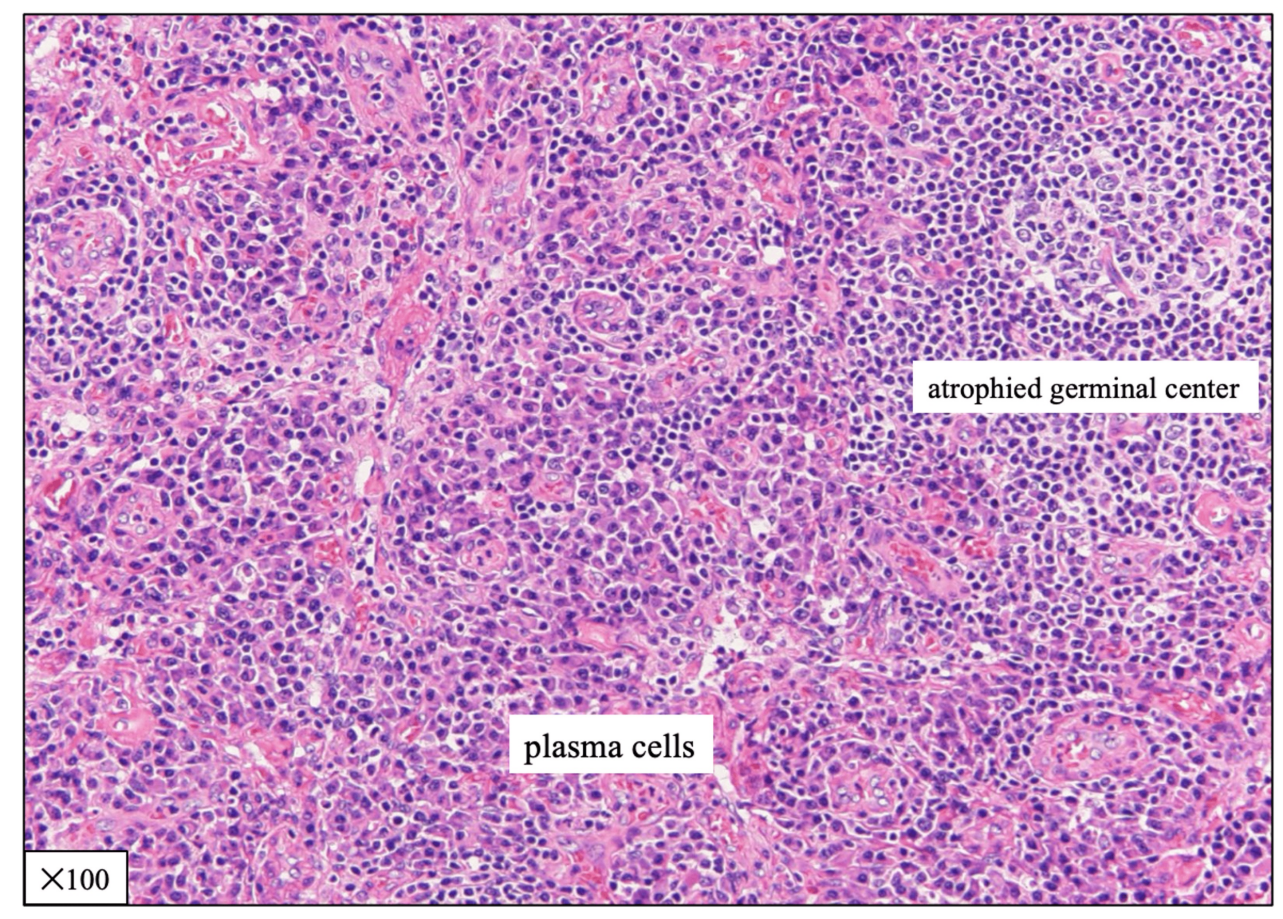
Histological findings of the biopsied right inguinal lymph node (hematoxylin and eosin stain) The histopathological finding revealed a prominent sheet-like increase of plasma cells in the interfollicular regions, and the atrophied germinal centers.

A right inguinal lymph node biopsy was performed, and diffuse plasmacytic proliferation in the interfollicular areas was revealed; histopathological findings suggest iMCD (Figure 2).

**Figure 3:**
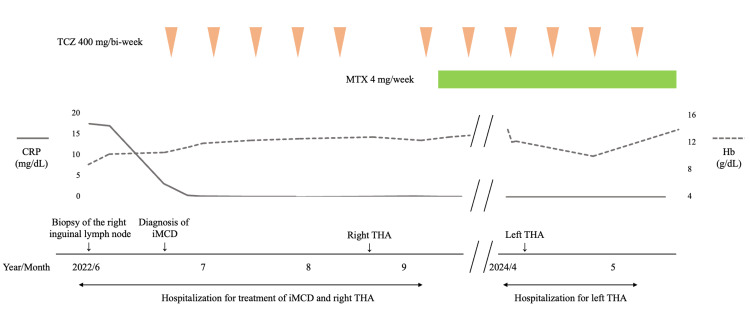
Clinical course of the patient CRP, C-reactive protein; Hb, hemoglobin; iMCD, idiopathic multicentric Castleman's disease; MTX, methotrexate; TCZ, tocilizumab; THA, total hip arthroplasty

IgG4 staining showed 62.3% of the plasma cells (IgG4/IgG positive), suggesting the presence of IgG4-related disease (IgG4-RD). However, the clinical course did not suggest IgG4-RD. Additionally, hemosiderin deposition, as shown in our case, is rare in IgG4-RD. An additional blood test showed no human herpesvirus 8 virus, but the serum IL-6 level was elevated at 153 pg/mL, and polyclonal hypergammaglobulinemia was observed (Table 1). She met the two major criteria (histological findings and lymph node enlargement), five laboratory minor criteria (high CRP titer, anemia, thrombocytosis, hypoalbuminemia, and hypergammaglobulinemia), and three clinical minor criteria (constitutional symptoms, liver enlargement, and leg edema), leading to a diagnosis of iMCD (plasma cell type) [2]. Malignant tumors, infectious diseases, and other autoimmune diseases were ruled out based on the clinical course and test results. The clinical course after admission is summarized in Figure 3.

**Figure 4:**
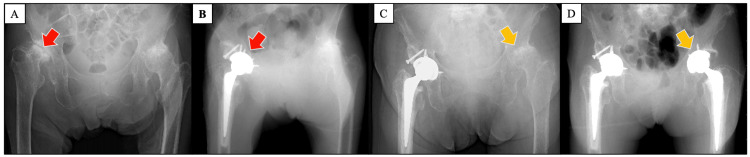
Pelvic X-ray transition (A) Pelvic X-ray at the time of admission to our hospital. (B) Two months after the initiation of TCZ; right THA was performed. Corresponding lesions are indicated by red arrows in figures (A) and (B), respectively. (C) One year after TCZ was initiated; left hip joint destruction had progressed. (D) Two years after the initiation of TCZ; left THA was performed. The corresponding lesions are marked with yellow arrows in figures (C) and (D), respectively. TCZ, tocilizumab; THA, total hip arthroplasty

Treatment with tocilizumab (TCZ) (anti-IL-6 receptor antibody) 400 mg biweekly was initiated, and the inflammatory markers resolved rapidly. Serum IL-6 level was elevated to 659 pg/mL two months after initiating TCZ. Even though the patient had no joint pain, joint destruction was found around the time of the iMCD diagnosis, and right total hip arthroplasty (THA) was performed (Figures 4A-4B).

**Figure 5:**
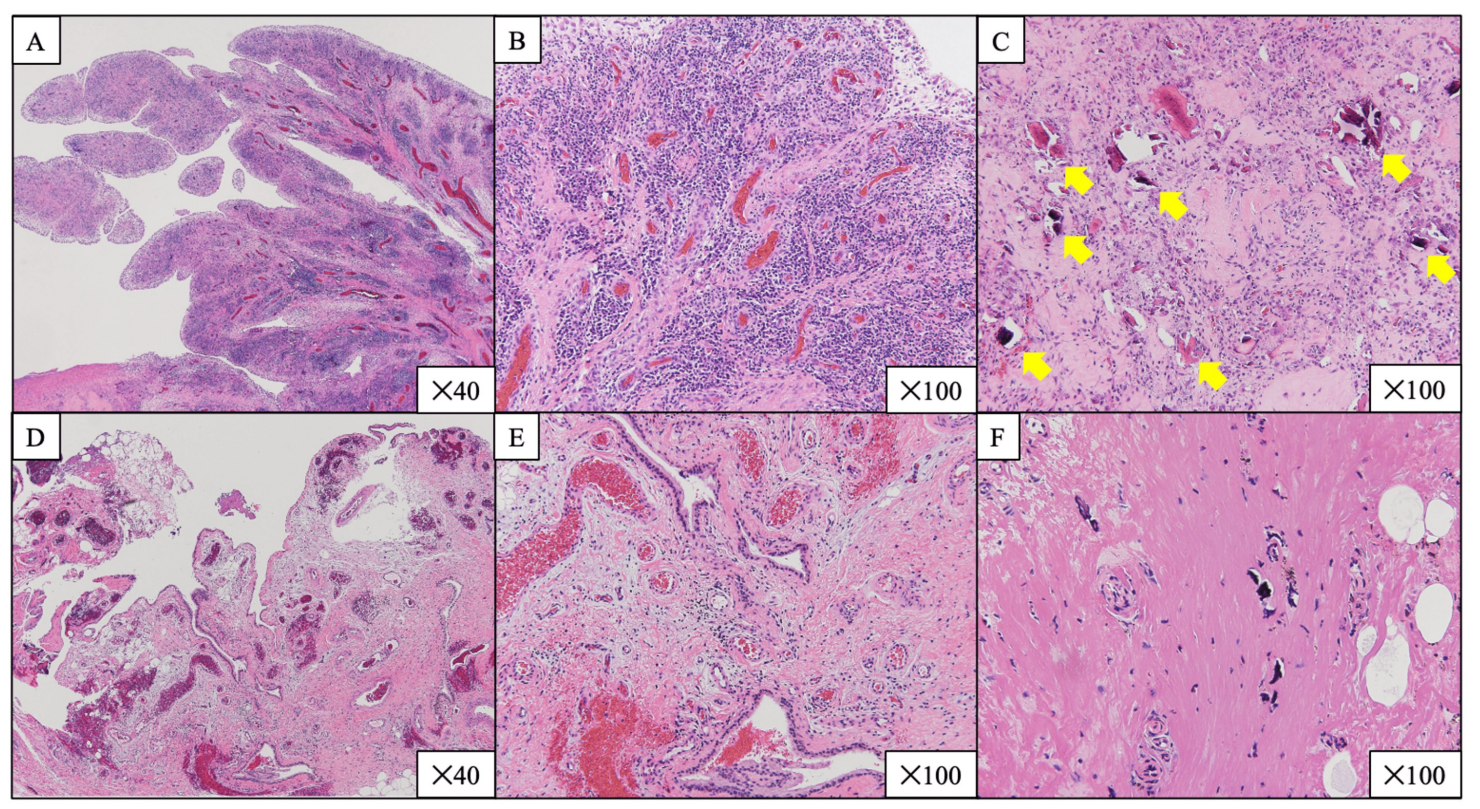
Histological findings of synovial tissue (A-C) Histological findings of synovial tissue at right THA (hematoxylin and eosin stain). (A) Synovial proliferation was marked. (B) Inflammatory cell infiltration was observed, especially of plasma cells. (C) Detritic synovitis was present; fine bone fragments were observed in the tissue (yellow arrows). (D-F) Histological findings of synovial tissue at left THA (two years after TCZ was initiated; hematoxylin and eosin stain). Synovial proliferation and inflammatory cell infiltration were reduced. TCZ, tocilizumab; THA, total hip arthroplasty

The synovial tissue showed detritic synovitis, suggesting bone destruction (Figures 5A-5C).

Synovial histological findings led to the diagnosis of iMCD complicated by RA-like synovitis. RA is one of the diseases that should be excluded in the diagnosis of iMCD [2]; therefore, the synovitis in our case was defined as RA-like synovitis and distinguished from RA synovitis. Methotrexate (MTX) was initiated to further enhance the effect of inhibiting bone destruction progression caused by RA-like synovitis. However, despite treatment with TCZ and MTX, the patient’s left hip joint bone deformity progressed over the following two years, so a left THA was performed (Figures 4C-4D). The histological findings of synovitis revealed low activity (Figures 5D-5F). Other joint destructions did not progress, and the patient had no recurrence of iMCD.

## Discussion

We experienced iMCD complicated by destructive synovitis (RA-like synovitis), which showed rapidly progressive joint destruction. Although TCZ improved inflammatory findings in the iMCD pathology, joint destruction progressed unexpectedly. This suggests that inflammatory pathways other than IL-6 led to joint destruction in our case. In Japan, RA is a relatively common disease; the estimated prevalence is approximately 6,500 people per 1 million [10], but iMCD is rare, affecting only 2.4-5.8 people per 1 million [11]. Previous reports of CD complicated with synovitis are shown in Table 2 [12-14].

**Table 2:** CD complicated with RA-like synovitis Ab, antibody; CCP, cyclic citrullinated peptide; CD, Castleman's disease; NA, not applicable; RA, rheumatoid arthritis; RF, rheumatoid factor

Case	Year	Sex	Age of onset	RF	Anti-CCP-Ab	Histrogical finding of synovitis
Ben-Chetrit et al. [12]	1989	Female	45	+	NA	NA
Otsuka et al. [13]	2019	Female	34	-	-	NA
Kondo et al. [14]	2022	Male	51	-	-	NA
Our case	2024	Female	69	+	-	+

In these cases, there is no mention of differences between CD and RA in the synovial tissue, nor of the progression of joint destruction. Thus, the differences in inflammatory pathology in the synovium between CD and RA remain unknown. To the best of our knowledge, this case is the first report of iMCD complicated with histological evidence of RA-like synovitis and rapidly progressive joint destruction, suggesting that destructive arthritis in iMCD may exhibit histological features similar to RA synovitis.

The co-existence of CD and RA-like synovitis was attributed to various factors, including T-cell activation and elevated cytokine levels in iMCD [13]. IL-6 plays a central role in both conditions [7]. In our case, serum IL-6 levels increased after TCZ administration. This mechanism is explained by TCZ occupying the IL-6 receptors, thereby inhibiting IL-6 clearance [15]. Additionally, increased serum IL-6 levels after TCZ administration differ between CD and RA, with a significant increase observed in CD but not in RA [15]. This difference can be attributed to CD's systemic and severe inflammatory pathology. While there is no evidence that IL-6 receptor-independent pathways lead to the increase of other inflammatory cytokines and bone destruction, it is possible that excessive serum IL-6 - as observed in inflammatory conditions more severe than RA - could secondarily induce pro-inflammatory cytokines that promote bone destruction. Moreover, pro-inflammatory cytokines other than IL-6 may play a central role in the inflammatory pathogenesis of some CD cases. IL-1β and tumor necrosis factor (TNF)-α are pro-inflammatory cytokines that stimulate IL-6 production via NF-κB signaling, and elevated levels of these cytokines have been reported in some iMCD patients [16,17]. In our patient, pro-inflammatory cytokines such as IL-1β and TNF-α, an upstream cytokine of IL-6, may have contributed to the pathogenesis of RA, leading to joint destruction.

Another inflammatory pathology common to CD and RA is the abnormal activation of mTOR [4,8,9]. The phosphatidylinositol-3-kinase (PI3K)/Akt/mTOR pathway is activated in the pathogenesis of iMCD [18]. Activating the PI3K/Akt/mTOR pathway suppresses autophagy in osteoclasts treated with hydrogen sulfide [19]. This finding suggests that the abnormal activity of mTOR in CD may lead to abnormal osteoclast activation in vivo. In RA, inflammatory cytokines interact with the mTOR signaling pathway, enhancing disease activity and increasing synovial inflammation, osteoclast differentiation, and joint destruction [20]. mTOR signaling regulates immune cell function, osteoclast differentiation, and survival. mTOR inhibition has shown promise in reducing synovial inflammation, osteoclast formation, and protecting against bone erosions and cartilage loss in experimental arthritis models [21]. Histologically, active mTOR signaling has been observed in synovial tissue from RA patients, particularly in synovial osteoclasts, highlighting its importance in disease pathogenesis [22]. In our case, it is hypothesized that the abnormal activation of mTOR in iMCD may have contributed to the progression of joint destruction. The interplay between these conditions possibly activates mTOR signaling pathways, which promote inflammation and osteoclast differentiation, leading to more severe joint destruction. This suggests a complex interaction between the underlying iMCD and RA-like synovitis, highlighting the pivotal role of mTOR in the pathogenesis of joint destruction. As mentioned above, although common bone metabolism dynamics have been demonstrated between iMCD and RA in highly inflammatory conditions, a sufficient investigation of differences in bone metabolism markers in actual clinical practice has not been conducted, and further research is required.

## Conclusions

In conclusion, our case highlights that the coexistence of iMCD and RA-like synovitis may exacerbate joint damage due to the amplification of inflammatory pathways. Further consideration is needed regarding the treatment strategy for cases of iMCD with destructive synovitis. As more cases are reported and further research is conducted, our understanding of the interaction between these pathologies will deepen.
